# Transcriptome Profiling of the Goose (*Anser cygnoides*) Ovaries Identify Laying and Broodiness Phenotypes

**DOI:** 10.1371/journal.pone.0055496

**Published:** 2013-02-06

**Authors:** Qi Xu, WenMing Zhao, Yang Chen, YiYu Tong, GuangHui Rong, ZhengYang Huang, Yang Zhang, GuoBing Chang, XinSheng Wu, GuoHong Chen

**Affiliations:** Key Laboratory of Animal Genetics & Breeding and Molecular Design of Jiangsu Province, Yangzhou University, Yangzhou, People's Republic of China; University of South Florida College of Medicine, United States of America

## Abstract

**Background:**

The geese have strong broodiness and poor egg performance. These characteristics are the key issues that hinder the goose industry development. Yet little is known about the mechanisms responsible for follicle development due to lack of genomic resources. Hence, studies based on high-throughput sequencing technologies are needed to produce a comprehensive and integrated genomic resource and to better understand the biological mechanisms of goose follicle development.

**Methodology/Principal Findings:**

In this study, we performed *de novo* transcriptome assembly and gene expression analysis using short-read sequencing technology (Illumina). We obtained 67,315,996 short reads of 100 bp, which were assembled into 130,514 unique sequences by Trinity strategy (mean size = 753bp). Based on BLAST results with known proteins, these analyses identified 52,642 sequences with a cut-off E-value above 10^−5^. Assembled sequences were annotated with gene descriptions, gene ontology and clusters of orthologous group terms. In addition, we investigated the transcription changes during the goose laying/broodiness period using a tag-based digital gene expression (DGE) system. We obtained a sequencing depth of over 4.2 million tags per sample and identified a large number of genes associated with follicle development and reproductive biology including cholesterol side-chain cleavage enzyme gene and dopamine beta-hydroxylas gene. We confirm the altered expression levels of the two genes using quantitative real-time PCR (qRT-PCR).

**Conclusions/Significance:**

The obtained goose transcriptome and DGE profiling data provide comprehensive gene expression information at the transcriptional level that could promote better understanding of the molecular mechanisms underlying follicle development and productivity.

## Introduction

China has the largest goose production in the world [1]–[3]. The goose is well known for its strong adaptability, rapid growth, rich nutrient content and low input requirement [4]. However, the goose industry development has been hindered by the goose’s strong broodiness and poor egg performance. In addition, the heritability of goose reproductive traits is low and conventional genetic improvement is difficult. Thus, it is important to understand the molecular mechanisms underlying its reproductive biology.

In recent years, the reproductive biology of the goose has attracted increasing attention. Kang B. (2009) identified some differentially expressed genes relevant to the reproduction of the geese from the prelaying to the egg-laying stage using suppression subtractive hybridization (SSH). These genes include estrogen receptor 1, estrogen receptor 2, follicle stimulating hormone receptor, prolactin receptor, ferritin H chain [5]. Guo J (2010) also found several differentially expressed genes between the laying and broodiness stage using a similar approach including the prolactin receptor, estrogen receptor α and anti-mullerian hormone receptor II [6]. DU XD (2009), ZHU P (2009) and WEI RH (2009) noticed that FSHβ PRLand PRLR mRNA are expressed regularly in the reproductive cycle [7]–[9]. Our team also consistently detected PIT-1 GH and PRL mRNA expression in the pituitary, hypothalamus and ovary by qRT-PCR [10]–[11]. As of February 1, 2012, there are about 430 EST and 7314 nucleotide sequences available for the goose in the NCBI database, and only 35 nucleotide sequences are relevant to reproduction. Obviously, these genetic data are insufficient for elucidating the molecular mechanism of productivity of the laying geese.

In the past years, next-generation high-throughput DNA sequencing techniques have provided fascinating opportunities in the life sciences and dramatically improved the efficiency and speed of gene discovery [12]. For example, Illumina sequencing technology offers millions of sequence reads from a single instrument run. This capacity permits gene expression profiling experiments with an improved dynamic range and considerable cost savings. Illumina sequencing of transcriptomes for organisms with completed genomes have confirmed that the relatively short reads production can be effectively assembled and used for gene discovery and comparison of gene expression profiles [13], [14]. Despite its wide use in studying animals of commercial value such as chicken, oyster, planarian and marine animals [15]–[18], high-throughput sequencing methods have not yet been applied to goose research.

In this study, we sequenced the goose transcriptome using Illumina technology and demonstrated the suitability of short-read sequencing for *de novo* assembly and annotation of genes expressed in a eukaryote without prior genome information. Furthermore, we compared the gene expression profiles of the goose between the laying and broodiness stages using a digital gene expression system. The assembled, annotated transcriptome sequences and gene expression profiles provide an invaluable resource for the identification of goose genes involved in follicle development and productivity.

## Materials and Methods

### Ethics Statement

All animal experiments were reviewed and approved by the Institutional Animal Care and Use Committee of School of Animal Science and Technology, Yangzhou University and performed in accordance with the Regulations for the Administration of Affairs Concerning Experimental Animals (China, 1988) and the Standards for the administration of experimental practices (Jiangsu, China, 2008). All surgery was performed according to recommendations proposed by European Commission (1997), and all efforts were made to minimize suffering of animals.

### Goose Rearing and Sample Preparation

The female Zhedong white geese were selected from 100 geese in the breeding farm of Jiangsu Lihua Animal Husbandry CO., LTD and raised according to the farm’s practice. During the experiment, geese were fed ad libitum with rice grain, supplemented with green grass or water plants whenever possible. The feed was given during daytime when the geese were released to an open area outside the house. The geese were exposed to natural lighting and temperature throughout this study. Ten geese were anesthetized with sodium pentobarbital and ovarian samples were obtained from laying geese and broodiness geese at 380 days of age. Ovarian samples, which comprised the whole ovary including the small and large yellow follicles. These rapidly removed, wrapped in freezing tube, frozen in liquid nitrogen, and then stored at −70°C until needed.

### cDNA Library Preparation and Illumina Sequencing for Transcriptome Analysis

Total RNA was extracted using Trizol reagent (Invitrogen, USA) following the manufacturer's protocol. RNA integrity was confirmed by the 2100 Bioanalyzer (Agilent Technologies). The samples for transcriptome analysis were prepared using Illumina's kit following manufacturer's recommendations. Briefly, mRNA was purified from 6 µg of total RNA (a mixture of RNA from laying and broodiness ovarian at equal ratio) using oligo (dT) magnetic beads. By using the fragmentation buffer, the mRNA was fragmented into short fragments (about 200 bp), then the first strand cDNA was synthesized with random hexamer-primer using the mRNA fragments as templates. Buffer, dNTPs, RNase H and DNA polymerase I were added to synthesize the second strand. The double-stranded cDNAs were purified with QiaQuick PCR extraction kit (Qiagen) and eluted with EB buffer for end repair and poly (A) addition. Finally, sequencing adapters were ligated to the 5′and 3′ends of the fragments. The fragments were purified by agarose gel electrophoresis and enriched by PCR amplification to create a cDNA library.

The cDNA library was sequenced on the Illumina sequencing platform (HiSeqTM 2000). The raw reads from the images were generated. After removal of the low quality reads, processed reads with an identity value of 95% and a coverage length of 100 bp were assembled using SOAP2 *de novo* software [19]. The clean reads were assembled with the Trinity program [20], and the Trinities were clustered using TGICL tools into unigenes [21]. The unigenes were used for BLAST search and annotation against an NCBI nr database using an E-value cut-off of 10^−5^. Functional annotation by gene ontology terms (GO;http://www.geneontology.org) was analyzed by Blast2Go software. The COG and KEGG pathways annotation was performed using BLAST all against Cluster of Orthologous Groups database and Kyoto Encyclopedia of Genes and Genomes database.

### Digital gene Expression (DGE) Library Preparation and Sequencing

The DGE libraries (three laying and three broodiness ovarian) were constructed as a transcriptome library. Only one adaptor was used as a sequencing primer (single -read). Each tunnel generated millions of raw tags with a length of 50 bp.

### Analysis and Mapping of DGE Reads

To map the DGE reads, the sequenced raw data were filtered to remove“dirty”raw reads containing the sequence of the adapter, more than 10% unknown bases or low quality reads (which are defined as reads having more than 50% bases with quality value ≤5). To annotation the reads, the clean reads were mapped to our transcriptome reference database, allowing no more than 2 nucleotide mismatch. The clean tags were designated as unambiguous clean tags. For gene expression analysis, the number of unambiguous clean tags for each gene was calculated and normalized to RPKM (Reads per kilobase transcriptome per million mapped reads).

A statistical analysis of the frequency of each read in the different cDNA libraries was performed to compare gene-expression in different tissue. Statistical comparison was performed with a custom written scripts using the method described [22]. FDR (false discovery rate) was used to determine the threshold of P value in multiple tests and analyses. We used FDR <0.001 as the threshold to judge the significance of gene expression difference. For GO and pathway enrichment analysis, we mapped all differentially expressed genes to terms in KEGG database and GO database, and looked for significantly enriched terms (following OE’s manual for RNA-Seq quantification).

### Quantitative Real-time PCR (qRT-PCR) Validation

Total RNA was extracted as described for the DGE library preparation and sequencing. The concentration of each RNA sample was adjusted to 1 µg/µl with nuclease-free water, and 2 µg of total RNA was reverse transcribed in a 20 µl reaction system using the AMV RNA PCR Kit (TaKaRa). The sequences of the specific primer sets are listed in Table 1. The glyceraldehyde-3-phosphate dehydrogenase gene (GAPDH) of goose was used as an internal control. The qRT-PCR was performed using the SYBR Premix Ex Taq Kit (TaKaRa) according to the manufacturer’s protocol. The results were normalized to the expression level of the constitutive GAPDH gene. A no template control (NTC) sample (nuclease free water) was included in the experiment to detect contamination and to determine the degree of dimer formation (data not shown). A relative quantitative method (ΔΔCt) was used to evaluate the quantitative variation.

**Table 1:** Primers used in qRT-PCR for validation of differentially expressed genes.

Genes for qRT-PCR	Primers of the gene for qRT-PCR	
cholesterol side-chain cleavage enzyme gene	Sense primer	5′-tgctgcaggactttgtgg -3′
	Antisense primer	5′-tggagaggatgcccatgt-3′
opamine beta-hydroxylas gene	Sense primer	5′-acgccaagatgaagccaga -3′
opamine beta-hydroxylas gene	Antisense primer	5′-agtgaatctcaaggcgcaga -3′
glyceraldehyde-3-phosphate dehydrogenase gene	Sense primer	5′-ggtggtgctaagcgtgtcat-3′
	Antisense primer	5′-ccctccacaatgccaaagtt-3′

## Results and Discussion

### Illumina Transcriptome Sequencing and Reads Assembly

**Figure 1:**
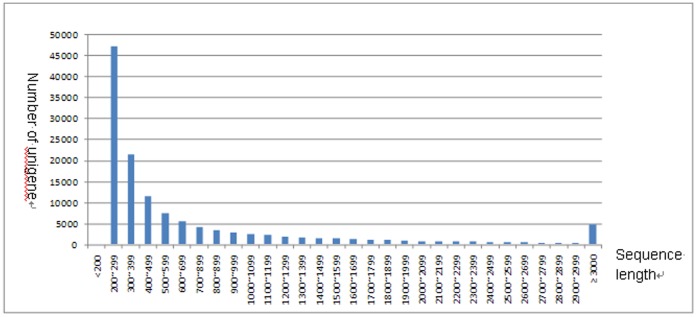
Histogram of the sequence length distribution of the significant matches. The x-axis indicates sequence sizes from 200 nt to≥3000 nt. The y-axis indicates the number of unigenes for every given sequence length. The results of the sequence–length matches (with a cut-off E-value of 1.0E−5) in the NCBI nr databases are greater among the longer assembled sequences.

To obtain an overview of the goose gene expression profile at the egg laying period, cDNA from ovarian tissues of laying/broodiness goose was sequenced by the Illumina sequencing platform. We obtained 67,315,996 short reads (accumulated length of 6,061,379,640 bp) by sequencing. Its GC content and Q20 were 49.82% and 96.02%, respectively. These reads were assembled with the Trinity software. Low-complexity and low-quality reads were filtered out resulting in 228,563 trinities. After clustering using the TGICL program, we obtained 130,517 unigenes (mean length: 753 bp) with the N50 of 1333 bp (Table S1). The size distribution indicated that the lengths of the 26,570 unigenes were more than 1000 bp (Fig. 1). At present, there are no standard criteria to evaluate the quality of transcriptome assemblies [23]. Researchers assess the quality of an assembly mostly by looking at the contiguity and accuracy of the assembly [24]. Due to the lack of genomic resources for goose, the goose mRNAs with full-length CDS from GenBank were considered as “gold standard” reference in our studies. We downloaded randomly 100 full length cDNA sequences of the goose (*Anser cygnoides*) from GenBank. We searched these cDNA sequences in the unigene database using BLASTN with a cut-off E-value of 10^−10^. The results showed a mean identity of 98.4% and query coverage of 69.5%. We also noticed that the mean length of unigene was longer than that from other research [25]–[26] mainly because of different assembly procedures. We assembled our data using Trinity, a new *de novo* transcriptome assembly package. Trinity could produce much more transcripts with length >200 bp [23]. The assembly results suggested that the unigenes data were highly reliable and cover most of the transcriptome sequences.

### Functional Annotation and Classification

**Figure 2:**
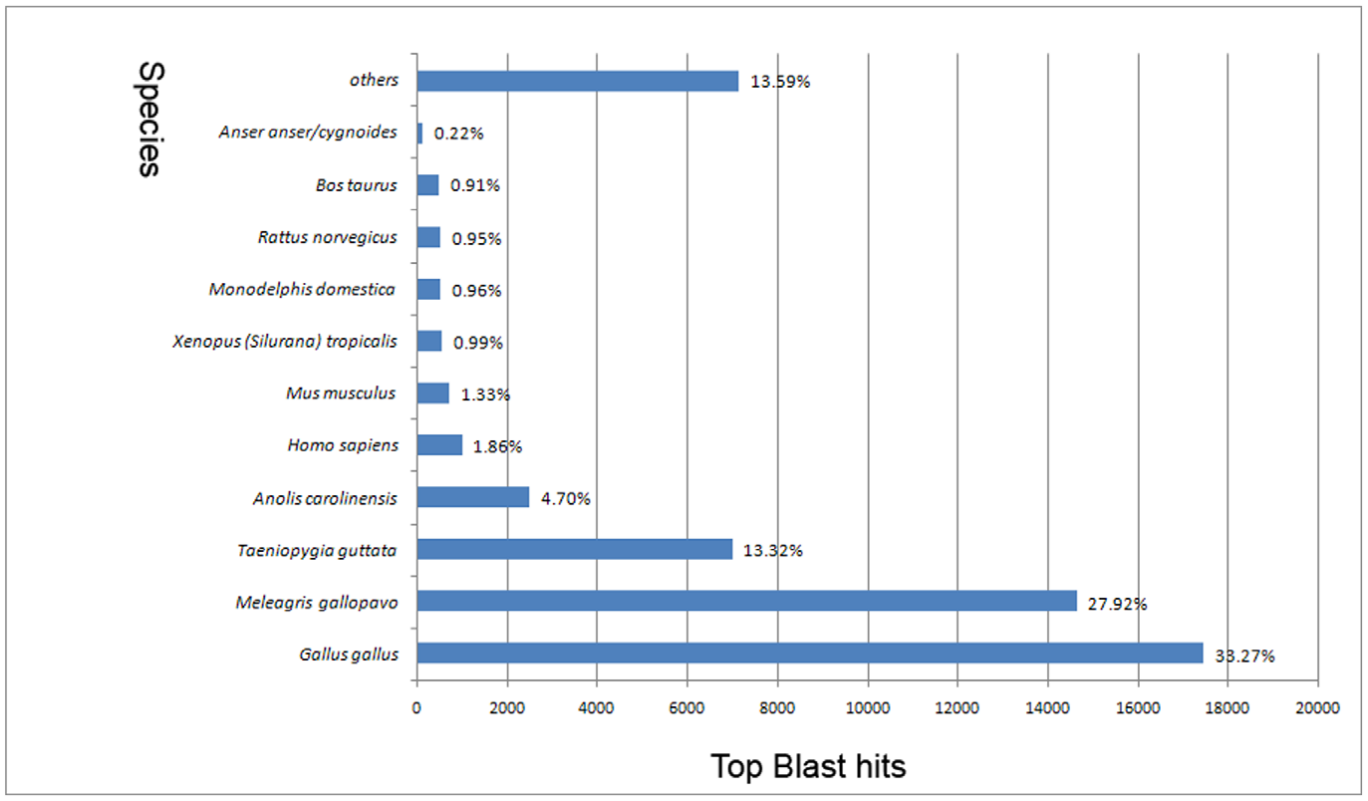
Top-Hit species distribution. 52,642 BLASTX-hit transcripts were calculated. More than 30% of the identified transcripts have the highest homology with *Gallus gallus*. Less than 0.3% of the top hits matched goose.

To annotate these unigenes, we searched the reference sequences using BLASTX against the protein database (non-redundant/nr and swissprot) with a cut-off E-value of 10^−5^. A total of 52,642 (40.33% of all distinct sequences) unigenes and 48,993 (37.54% of all distinct sequences) unigenes were obtained as significant hits using the respective databases (Table 2). However, there were still a large number of sequences (77,478, 59.36%) without BLASTX hits. Most of these sequences were short fragments, and some of the sequences without BLASTX hits might be new genes or non-coding RNA sequences. Among the 52,642 BLASTX-hit transcripts, only 0.22% (113) of the top matched hit goose itself (Figure 2), which could be explained by the limited number of the goose protein sequences that were currently available in the NCBI database. Most of the identified unigene showed the highest homology with chicken (17,444, 27.92%).

**Table 2:** All-in-one list of annotations.

Annotation database	No of annotation	Percent of annotation (%)
total unigene	130517	100
Nr	52642	40.33344
Swissprot	48993	37.53764
COG	18639	14.2809
KEGG	39689	30.40907
GO	14474	11.08974
Unknown	77478	59.36238

### Gene Ontology (GO) and Clusters of Orthologous Groups (COG) Classification

**Figure 3:**
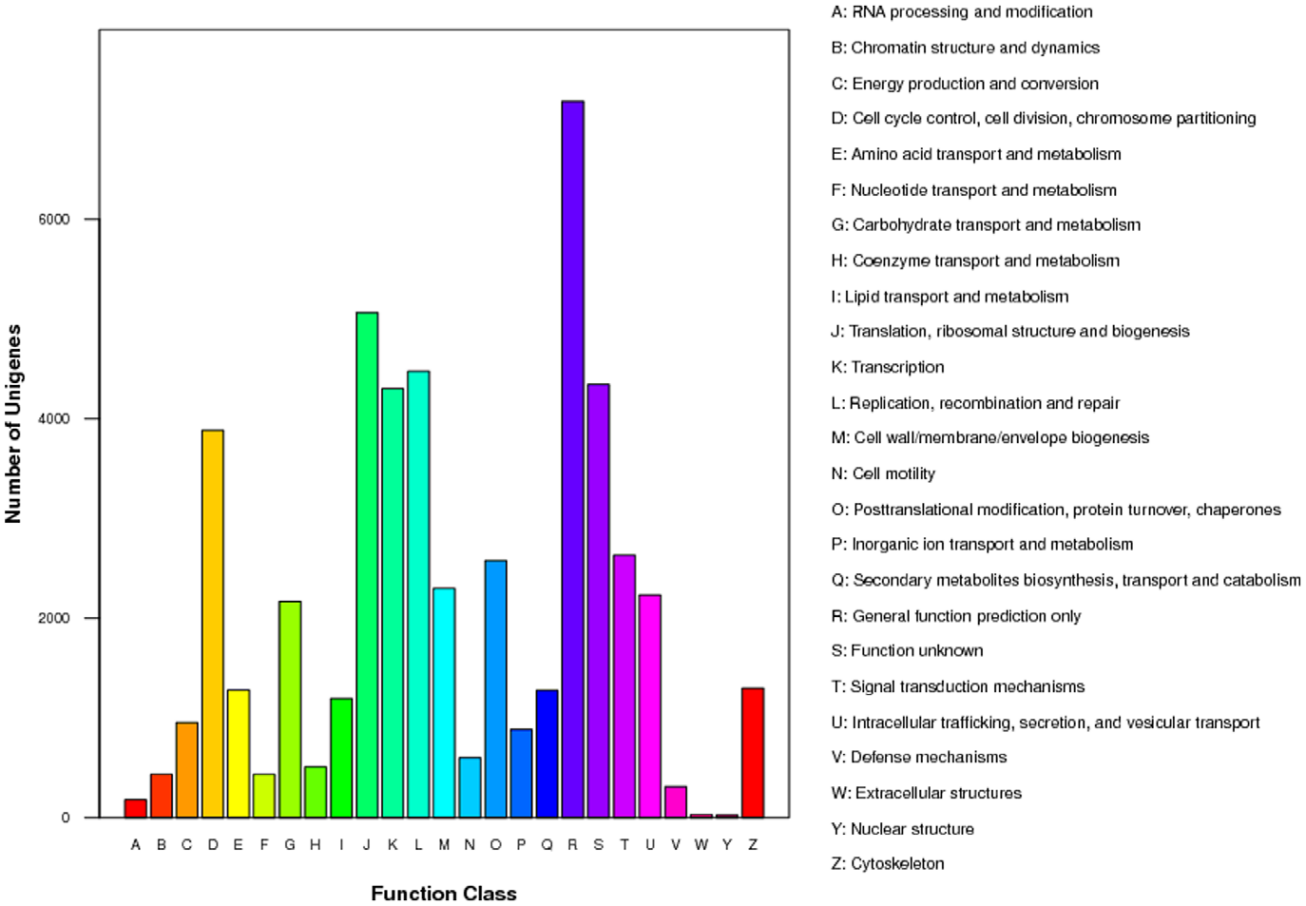
Histogram presentation of clusters of orthologous groups (COG) classification. 18,639 sequences were grouped into 25 COG categories.

To evaluate the completeness of our transcriptome library and the effectiveness of our annotation process, we searched the annotated sequences for the genes involved in COG classifications [27]. Unigenes were aligned to the COG database to predict and classify possible functions. A total of 18,639 sequences had a COG classification (Figure 3).

Among the 25 COG categories, the cluster for ‘General function prediction’ represented the largest group (7182, 14.20%) followed by ‘Translation, ribosomal structure and biogenesis’ (5064, 10.01%) and ‘Replication, recombination and repair’ (4477, 8.85%). Extra-cellular structures (29, 0.57%) and Nuclear structure (26, 0.51%) represented the smallest groups (Figure 3).

To further survey the biological pathways that are active in the goose, we mapped the annotated sequences to the reference canonical pathways in the Kyoto Encyclopedia of Genes and Genomes (KEGG) [28]. We assigned 39,689 sequences to 234 KEGG pathways, most represented by the unique sequences were “Metabolic pathways” (3913 members); “Regulation of actin cytoskeleton” (2121 members) and “Focal adhesion” (1988 members). Intriguingly, we also noticed several signaling networks of reproductive biology, such as “Oocyte meiosis” and “Calcium signaling pathway”.

**Figure 4:**
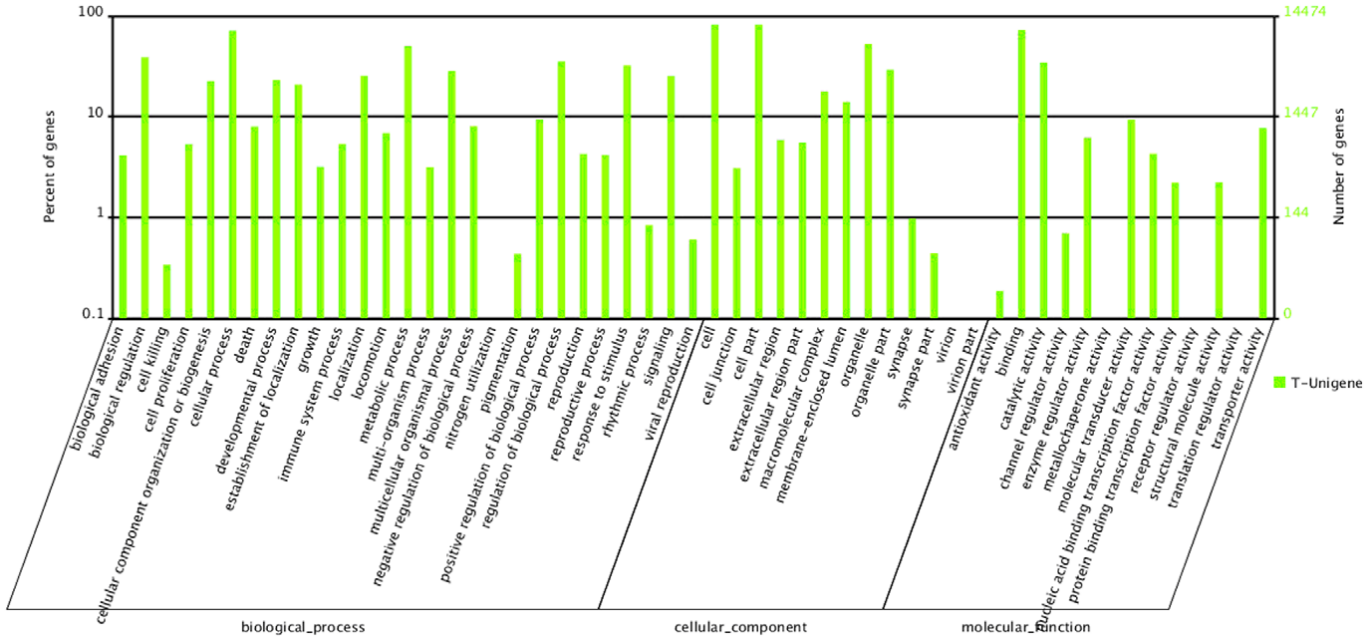
Histogram presentation of Gene Ontology classification. The results are summarized in three main categories: biological process, cellular component and molecular function. The y-axis on the right indicates the number of genes in a category. The y-axis on the left indicates the percentage of a specific category of genes in that main category.

Finally, we obtained the Gene Ontology (GO) functional annotation with the nr annotation [29]. GO assignments were used to classify the functions of the predicted goose genes. In total, 14,474 sequences could be categorized into 53 functional groups (Figure 3). The terms “Cellular process”, “Cell” and “Binding” were most represented in the three main categories of cellular component, molecular function and biological process, respectively (Figure 4). As expected, “reproduction” and “reproductive process” were found in the categories of biological process. We also noticed that only a few of genes were associated with terms such as “nitrogen utilization”, “virion part” and “metallochaperone activity” (Figure 3). Results from our analysis of the goose transcriptome further verified our idea that these annotations provided a valuable resource for investigating specific processes, functions and pathways for reproductive biology research in goose.

### Digital Gene Expression (DGE) Library Sequencing

**Figure 5:**
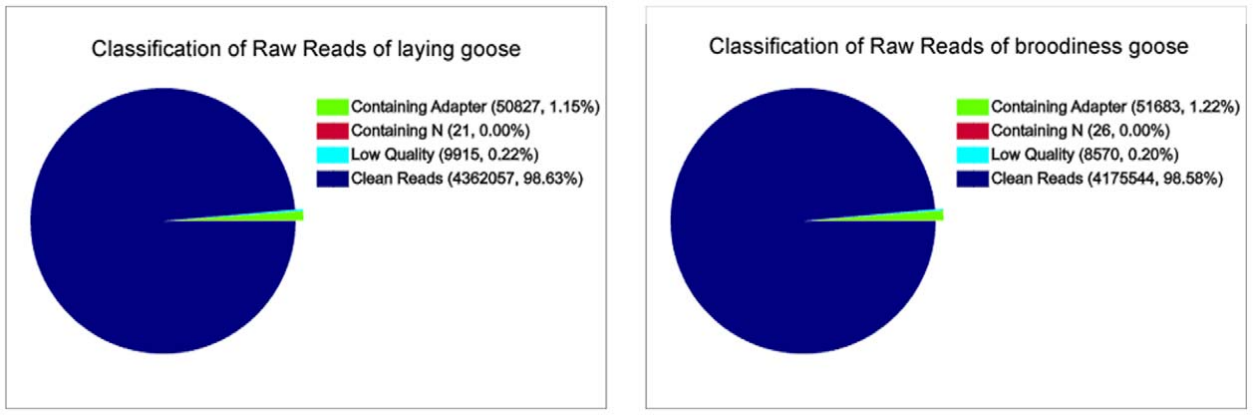
Different components of the raw reads in each sample. The percentages of tags containing N, adaptors, clean tags and low quality reads. The numbers in parentheses indicate the percentage of each type of read among the total raw reads.

To characterize the digital gene expression profiles in the goose, the two DGE libraries (ovarian tissues of laying and broodiness goose) were constructed and sequenced using Illumina deep sequencing platform. About 4.42 and 4.24 million raw tags were generated in each library (Figure 5). About 98% of the raw reads passed the filter, resulting in 4.36 and 4.17 million clean reads in the two respective libraries. The clean reads mapped to gene with no more than two mismatches were about 3.5 to 3.7 million in the two respective libraries, in which about 70% reads were perfect matched (Table 3).

**Table 3:** Statistics of DGE sequencing.

Map to gene	tissues of laying goose		tissues of broodiness goose	
	Number	Percentage	Number	Percentage
Total reads	4362057	100.00%	4175544	100.00%
Total basepairs	213740793	100.00%	204601656	100.00%
Total mapped reads	3701723	84.86%	3549513	85.01%
Perfect match	3057906	70.10%	2956764	70.81%
< = 2 mismatch	643818	14.76%	592749	14.20%
Unique match	2059890	47.22%	1953130	46.78%
Multi-position match	1641833	37.64%	1596383	38.23%
Total unmapped reads	660334	15.14%	626031	14.99%

### Mapping Sequences to the Reference Transcriptome Database

**Figure 6:**
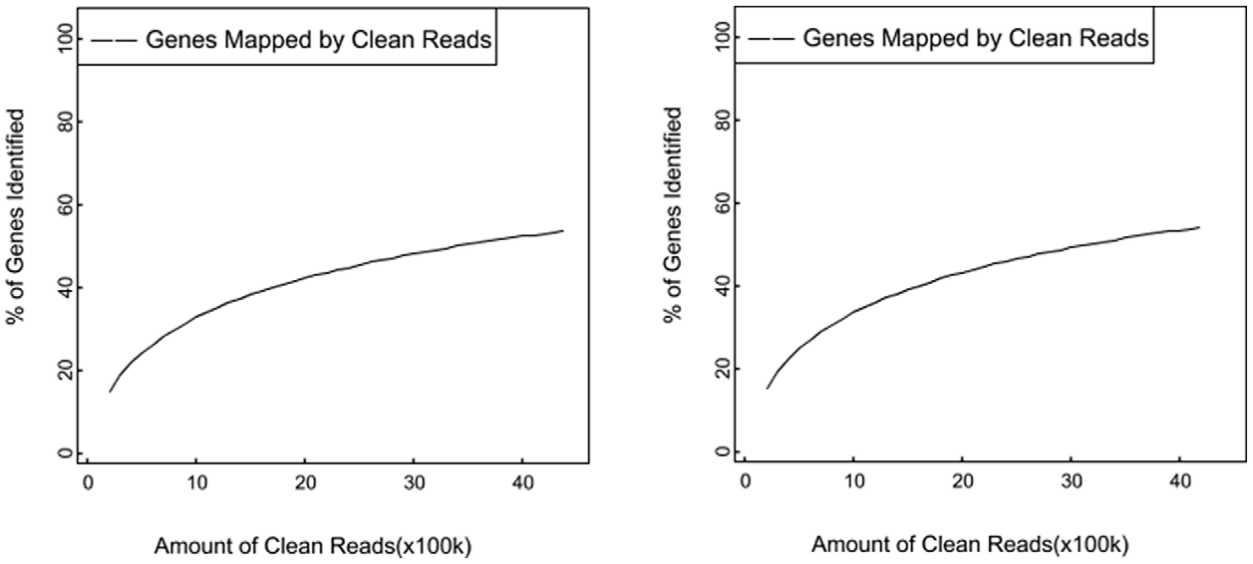
Sequencing saturation analysis. X-axis is number of clean reads, Y-axis is the percentage of identified genes. When the number of clean reads reaches about 4 M or higher, the number of detected genes almost ceases to increase. The left figure show the DGE of laying goose, the right figure show the DGE of broodiness goose.

To reveal the molecular events behind the DGE profiles, we mapped the tag sequences of the two DGE libraries to our transcriptome reference database generated in the above-mentioned Illumina sequencing. This reference database contains 130,517 distinct unigene. 72,961 and 73,641 unique genes had a mapped gene in the reference database. To confirm whether the number of detected genes increases proportionally to sequencing amount (total clean reads number), saturation analysis was performed. Figure 6 showed a trend in saturation where the number of detected genes almost ceased to increase when the number of reads reaches 4 million.

### Analysis of Gene Differential Expression

To discover the genes displaying a significant difference in expression during different laying egg stages, the gene expression levels were calculated using the RPKM method [30] (Reads per kilobase transcriptome per million mapped reads). A total of 86326 entities were detected between the laying and broodiness of ovarian libraries. Filtered with FDR≤0.001 and |log2Ratio|≥1 [31], we identified 572 significantly differential expression genes with 294 up-regulated and 278 down-regulated genes (Table S2 ). We observed that more than 35% of the highly expressed genes were orphan sequences – no homologs were found in the NCBI database. Those might be an indication that these sequences were expressed specifically in the goose ovarian tissues. In addition, we also noticed some reproductive genes including growth differentiation factor 9 and vasoactive intestinal polypeptide receptor 2 genes. Compared with the previous studies detected by SSH [5]–[6], we did not detect FSHβ, PRL and PRLR genes expressed differentially. Actually, DGE provides an unbiased methodology to investigate the expression pattern for each gene based on deep sequencing, while SSH often presents false positives.

### Functional Annotation of Differentially Expressed Genes

**Figure 7:**
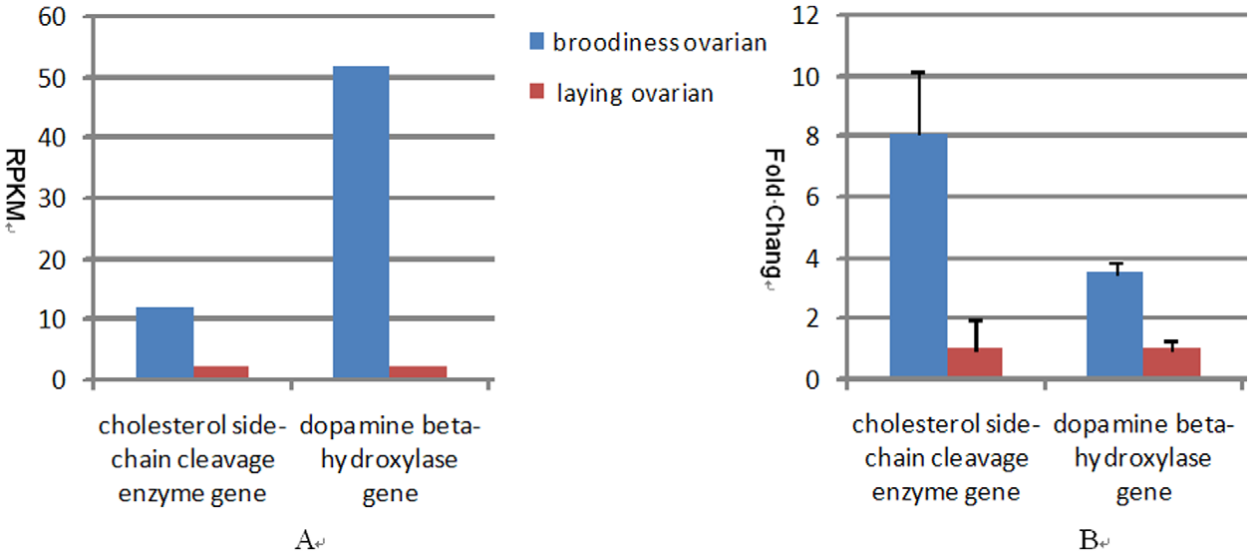
Analyses of the differentially expressed genes during the goose laying stage. (A) The gene expression levels of cholesterol side-chain cleavage enzyme gene and dopamine beta-hydroxylase gene were determined by calculating the number of unambiguous reads for each gene and then normalizing to RPKM (Reads per kilobase transcriptome per million mapped reads). (B) Gene expression data obtained by qRT-PCR analysis. Expression ratios of the two genes in ovarian samples from laying and broodiness geese.

To understand the functions of the differentially expressed genes, we carried out GO functional enrichment and KEGG pathway analysis. According to the GO classification, most of the gene sets demonstrated down-regulated expression in the ovarian tissues library of laying goose, and associated with reproduction process, i.e. norepinephrine metabolic process, steroid metabolic process, steroid biosynthetic process, and reproductive process (Table S3). Among the genes with the KEGG pathway annotation, 355 differentially expressed genes were identified between the laying and broodiness ovarian libraries (Table S4). Notably, specific enrichment of genes was observed for pathways involved in reproduction regulation, such as steroid hormone biosynthesis, Wnt signaling pathway, Calcium signaling pathway, GnRH signaling pathway and Oocyte meiosis. In a comparison between GO functional enrichment and KEGG pathway analysis, we found some gene in the common pathway(steroid hormone biosynthesis), such as cholesterol side-chain cleavage enzyme gene. Additionally, we also found several genes in one pathway of DEGs, such as the dopamine beta-hydroxylas, adenylyl cyclase-associated protein 2 gene and so on. To further evaluate our DGE data, we analyzed the expression level of some genes. As shown in Figure 7A, both cholesterol side-chain cleavage enzyme gene and dopamine beta-hydroxylas gene were highly expressed in the broodiness goose. To confirm the results of DGE analysis, the expression levels of the two genes were measured in laying and broodiness geese by qRT-PCR. The qRT-PCR data for the two genes were consistent with those obtained by DGE expression profiling (Figure 7B). Previous research on chicken and pig confirmed that the two genes are closely related to follicle development and reproductive performance [32]–[34]. Therefore, we believed that steroid hormone biosynthesis gene and dopamine beta-hydroxylas gene played an important role in follicle development and productivity of goose.

### Conclusion

This study investigated the transcriptome profile of the ovarian tissues from the laying/broodiness goose using the Illumina RNA-seq and DGE deep-sequencing technologies. We generated 130,514 unique sequences with 52,642 sequences with a cut-off E-value above 10^−5^. These findings provided a substantial contribution to existing sequence resources for the goose and would certainly accelerate follicle development research on goose. Additionally, we demonstrated the feasibility of using Illumina sequencing-based DGE system for gene expression profiling and provided new data for functional studies of genes involved in goose productivity.

## Supporting Information

Table S1 **Annotation for the unigenes.**
(ZIP)

Table S2 **The differentially expressed genes in the two libraries.**
(XLSX)

Table S3 **Significantly enriched GO terms in DEGs.**
(XLSX)

Table S4 **Pathway enrichment analysis of DEGs.**
(XLSX)
